# Two Faces of Glutaminase GLS2 in Carcinogenesis

**DOI:** 10.3390/cancers15235566

**Published:** 2023-11-24

**Authors:** Joanna Buczkowska, Monika Szeliga

**Affiliations:** Department of Neurotoxicology, Mossakowski Medical Research Institute, Polish Academy of Sciences, 5 Pawińskiego Str., 02-106 Warsaw, Poland; jbuczkowska@imdik.pan.pl

**Keywords:** glutaminase GLS2, tumor promoter, tumor suppressor, glutamine metabolism, oxidative stress, epithelial-mesenchymal transition, apoptosis, ferroptosis

## Abstract

**Simple Summary:**

Glutaminase GLS2 is an enzyme that metabolizes glutamine, one of the main sources of energy and building blocks in tumor cells. In some types of cancer, this enzyme is overexpressed and fuels malignancy; however, there are some types of cancer in which GLS2 plays a role opposite to that of a tumor suppressor. The mechanisms behind the GLS2-mediated inhibition of tumorigenesis are complex and context-dependent. In this review, we report both the pro- and anti-tumorigenic properties of this protein.

**Abstract:**

In rapidly proliferating cancer cells, glutamine is a major source of energy and building blocks. Increased glutamine uptake and enhanced glutaminolysis are key metabolic features of many cancers. Glutamine is metabolized by glutaminase (GA), which is encoded by two genes: *GLS* and *GLS2*. In contrast to isoforms arising from the *GLS* gene, which clearly act as oncoproteins, the role of *GLS2* products in tumorigenesis is far from well understood. While in some cancer types *GLS2* is overexpressed and drives cancer development, in some other types it is downregulated and behaves as a tumor suppressor gene. In this review, we describe the essential functions and regulatory mechanisms of human GLS2 and the cellular compartments in which GLS2 has been localized. Furthermore, we present the context-dependent oncogenic and tumor-suppressor properties of GLS2, and delve into the mechanisms underlying these phenomena.

## 1. Introduction

Enhanced growth and the proliferation of cancer cells lead to increased demand for energy and biomacromolecules. To meet this requirement, cancer cells rewire their metabolism. One of the classical examples of the metabolic pathway being altered by cancer is the Warburg effect, defined as enhanced glucose uptake and lactate production, carried out regardless of oxygen availability [1]. In addition to glycolysis, cancer cells metabolize large amounts of glutamine (Gln) to sustain proliferation and grow unhindered. Gln is catabolized to glutamate (Glu) and further converted to α-ketoglutarate (α-KG), which enters the tricarboxylic acid (TCA) cycle to generate energy and provide precursors for proteins, nucleotides, and lipids. Gln-derived Glu is used for glutathione (GSH) synthesis to maintain redox homeostasis. Additionally, Gln is involved in the regulation of cell signaling. The aforementioned pleiotropic role of Gln in carcinogenesis has been exhaustively addressed in several reviews [2,3]. A growing body of evidence shows that Gln deprivation inhibits the survival of cancer cells [4,5,6,7,8,9] and indicates that Gln addiction is an important therapeutic target in a wide variety of cancer types.

**Figure 1:**
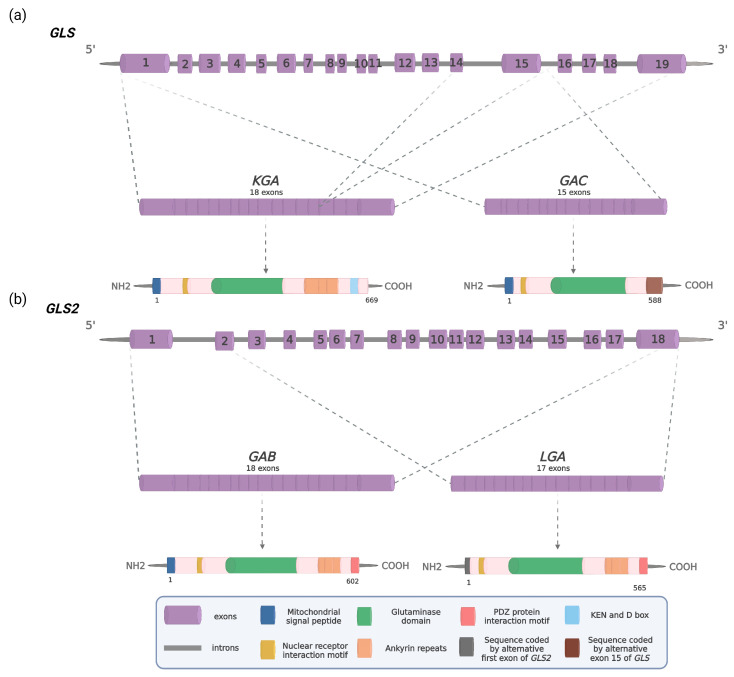
Schematic illustration of human glutaminases: (**a**) The *GLS* gene and two alternative transcripts and proteins KGA and GAC; (**b**) The *GLS2* gene and two alternative transcripts and proteins GAB and LGA. Introns are depicted as a solid grey line and exons as numbered purple cylinders. Dashed gray lines indicate the exons forming *KGA*, *GAC*, *GAB*, and *LGA* mRNA transcripts, respectively. Dashed gray arrows indicate KGA, GAC, GAB, and LGA proteins with defined main signature sequences and motifs. Adapted with permission from [13]. 2016, SNCSC. Adapted with permission from [14]. 2015, Elsevier. Created with BioRender.com.

Gln is hydrolyzed to Glu and ammonia by glutaminase (GA, EC 3.5.1.2). Human GAs are encoded by two genes, *GLS* and *GLS2*, located in chromosome 2 and chromosome 12, respectively [10]. The *GLS* gene codes for kidney-type GA (KGA) and glutaminase C (GAC) originate through alternative splicing of a single pre-mRNA [11]. GAC is identical to KGA except for the C-terminal region encoded by exon 15, which is missing in KGA (Figure 1) [12,13,14]. Of the two isozymes, GAC shows higher enzymatic activity, which is most likely related to its unique C terminus [15]. In fact, overexpression of the *GLS* gene has been documented in several cancer types of different origin and, in most cases, GAC is the predominant GLS isoform [15,16,17,18,19,20,21,22,23,24]; moreover, the silencing of *GLS* expression reversed the malignant phenotype of cancer cells both in vitro and in vivo, proving the important role *GLS* products play in promoting tumorigenesis [25,26,27,28,29,30]. These findings clearly indicate that GLS proteins are attractive therapeutic targets. Indeed, several GLS inhibitors have demonstrated efficacy in preclinical cancer models and some of them have entered clinical trials [31,32].

In contrast to GLS isoforms, which clearly act as oncoproteins, the role of *GLS2* products in tumorigenesis is far from well understood. While in some cancer types *GLS2* is overexpressed and drives cancer development, in some other types it is downregulated and behaves as a tumor suppressor gene. In this article, we briefly discuss the essential functions and regulatory mechanisms of human GLS2. Furthermore, we review its tumor promoting and suppressing properties, and delve into the mechanisms underlying these phenomena.

## 2. The *GLS2* Gene and Its Products

The two *Gls2* products, both protein and cDNA, were first isolated from rat liver [33,34,35], which is why this glutaminase was named liver-type glutaminase (LGA). Compared to the enzymes encoded by the *Gls* gene, rat hepatic LGA protein has a higher Michaelis constant (Km) for Gln and is fully activated at lower concentrations of inorganic phosphate. Furthermore, it is not inhibited by Glu and is activated by ammonia [33,34].

The human *GLS2* gene spans more than 18.1 kb and consists of 18 exons [10,36]. To date, two transcripts arising from this gene have been identified. The canonical transcript contains all 18 exons and has an 1806-base open reading frame (ORF) encoding a 602-residue protein. It was cloned for the first time from human breast cancer cells [37] and later termed GAB in order to distinguish it from the LGA isoform encoded by a short transcript of the same gene [38]. The *LGA* transcript has a 1698-base ORF encoding a putative protein of 565 amino acids; it arises from an alternative transcription initiation occurring at an alternative promoter located in intron 1 (Figure 1).

**Figure 2:**
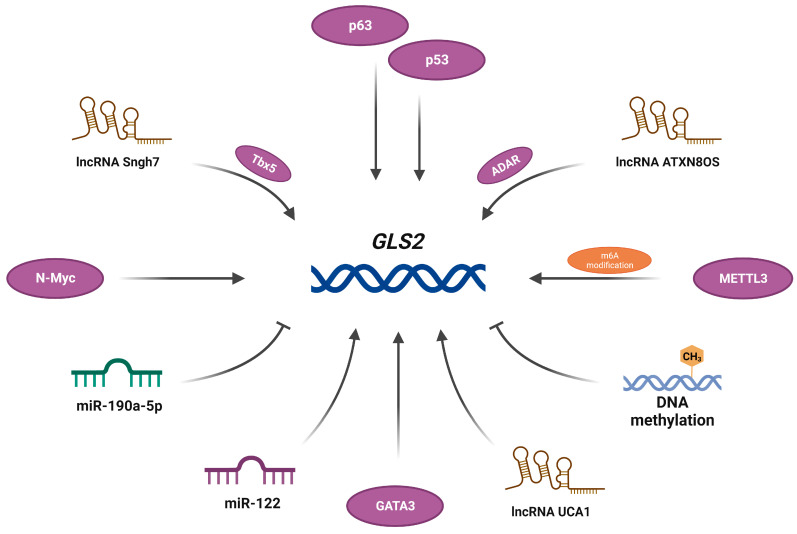
Molecules which regulate *GLS2* expression. Based on [39,41,42,43,44,45,46,47,48,49,50,51,52,53,54]. Created with BioRender.com.

Both canonical and alternative *GLS2* promoters contain CAAT- and TATA-like boxes, a Specificity protein 1 (Sp1) site and a tumor protein 53 (p53) site [36,39,40]. Indeed, p53 has been shown to induce *GLS2* expression in human lung cancer and glioblastoma cells [39,41]. It is noteworthy that the transcription factor p63 belonging to the p53 family binds directly to the p53/p63 consensus DNA-binding sequence within the *GLS2* promoter region and induces transcription of this gene [42]; moreover, Xiao and colleagues identified a Myc-binding site within the first intron of *GLS2* and documented a direct activation of the transcription of this gene by N-Myc in *MYCN*-amplified neuroblastoma cells [43]. Despite the similarities noted above, canonical and alternative promoters differ with regard to other regulatory elements: the *GAB* promoter has an RREB site and an extremely high content of CpG islands, while the *LGA* promoter contains binding sites for Activator protein 1 (AP-1) and GATA-binding factor 1 (GATA-1), and it lacks a CpG island [36,40]. In fact, *GLS2* promoter methylation, leading to a deficit in its product, was observed in liver and colon cancer [44,45], glioblastoma [46], and basal-subtype breast tumors [47]. Interestingly, in luminal-subtype breast cancer cells, *GLS2* expression is upregulated via GATA-binding factor 3 (GATA3) [47], indicating that its expression pattern differs not only between tissues but, also, between cancer subtypes affecting the same tissue. As well as DNA methylation, RNA methylation is another epigenetic mechanism implicated in the regulation of *GLS2* expression. Methyltransferase-like protein 3 (METTL3) is a component of a methyltransferase complex that catalyzes the addition of N6-methyladenosine (m6A) to mRNA [48]. Recently, METTL3-mediated m6A modification has been proven to enhance the stability of the *GLS2* transcript in esophageal squamous cell carcinoma cells [49]. In addition, accumulating evidence suggests the influence of non-coding RNA on *GLS2* expression. Long non-coding RNA urothelial carcinoma-associated 1 (lncRNA UCA1) regulates the expression of *GLS2* by interfering with miR-16 in bladder cancer cells [50]. It has been proven that miR-190a-5p directly targets *GLS2* in human cardiomyocytes [51]. In addition, an indirect impact of lncRNA small nucleolar RNA host gene 7 (Snhg7) on the expression of *GLS2*, through T-box transcription factor 5 (Tbx5), has been documented [52]. In glioblastoma cells, another lncRNA, ATXN8 Opposite Strand (ATXN8OS), has been shown to stabilize the *GLS2* transcript by recruiting the RNA-binding protein (RBP) ADAR [53]. It is noteworthy that, in human hepatocellular carcinoma, *GLS2* expression positively correlates with the level of miR-122, which inhibits *GLS* expression; moreover, *Gls2* expression is lower in liver tissues from miR-122 knockout mice compared to wild-type animals [54]. Molecules which have been identified as regulators of *GLS2* transcription are presented in Figure 2.

Although *GLS2* was originally thought to be expressed exclusively in adult liver [37], several later studies proved its expression in extrahepatic tissues such as brain, pancreas, and human cancer cells, and cells of the immune system [17,37,55,56]. It is worth noting that western blot analysis of human neuroblastoma SHSY-5Y cell lysates revealed two protein bands with molecular masses compatible with GAB and LGA, confirming the existence of two *GLS2* products [40]. Interestingly, human GAB expressed in baculovirus presented low affinity for Gln and low phosphate dependence, as expected for a GLS2 protein, but it was inhibited by Glu, demonstrating a kinetic behavior typical of GLS isoforms. Furthermore, human GAB was scarcely activated by ammonia, unlike the GLS2 enzyme isolated previously from liver [57]. It should be emphasized that the GAB enzyme contains the sequence encoded by exon 1 and the first six amino acids of exon 2, which is not present in the LGA enzyme. It is therefore tempting to speculate that this region is essential for Glu inhibition and ammonia activation; moreover, it may be important for the trafficking of GAB to a particular cellular compartment.

The N-terminal region of GAB contains a mitochondrial targeting sequence [37] and, indeed, GLS2 protein was detected in the mitochondrial fractions of human breast cancer cells, both wild-type an ectopically expressing *GLS2* [47]. Similarly, mitochondrial localization of the exogenous GLS2 protein was found in human lung cancer, hepatoma, and neuroblastoma cells transfected with *GLS2*-expressing vector [39,58]. Importantly, in several human tumor cells, GLS2 was detected mainly in mitochondria but, in approximately 20% of these cells, it was localized in nuclei; moreover, a significant increase in the amount of GLS2 in nuclei was observed in either SH-SY5Y or HepG2 cells treated with the differentiation agent phorbol 12-myristate 13-acetate (PMA) or T98G glioblastoma cells transfected with the full *GLS2* ORF [58]. These findings are consistent with previously reported nuclear localization of GLS2 in the rodent and monkey brain [59,60]. To date, the mechanism by which this protein enters the nucleus has not been identified. The GLS2 sequence lacks a classical nuclear localization signal, but has some other motifs that could be essential for the nuclear import. It possesses the sequence LGDLL in exon 1, which conforms to the LXXLL motif that facilitates the interaction of different proteins with nuclear receptors [59]. Furthermore, GLS2 was shown to interact with some PDZ domain-containing proteins through the ESMV motif in the C-terminus [61]. Given that several PDZ domain-containing proteins have been reported to localize to the nucleus, GLS2 might reach this compartment through interactions with such proteins [59,62]. Additionally, ankyrin repeats have been detected in the C-terminus of GLS2. Ankyrin-repeat motifs are crucial for the nuclear localization of several proteins; therefore, it is possible that they substitute for a classical nuclear localization signal in GLS2 [62]. Interestingly, in PMA-treated HepG2 cells, a perinuclear GLS2-positive staining has been detected. The perinuclear zone was enriched with vesicle-associated membrane protein 8 (VAMP8)-containing transport vesicles, which overlapped with the GLS2 staining, suggesting that the nuclear transport of GLS2 could be vesicle mediated [58]. It cannot be ruled out that GLS2 enters the cell nucleus through hypusination, a rare post-translational modification that converts lysine into hypusine (N^ε^-(4-amino-2-hydroxybutyl)lysine), so far identified only in eukaryotic translation initiation factor 5A (eIF5A). The significance of this modification is still far from well understood, but increasing evidence points to its role in protein localization and cellular signaling [63]. Hypusinated lysine was detected in the unfolded segment of GLS2, and the location of this modified residue suggests that it is exposed to the external medium [58]. Given that such exposed hypusine has proven to be necessary for the interaction between eIF5A and Xpo4 exportin, and evidently enables nucleocytoplasmic shuttling [64], this modification might be involved in the nuclear import of GLS2.

It is noteworthy that, in human neutrophils, GLS2 has been shown to be present in the cell surface. In intact neutrophils, GLS2 was secreted from the secondary granules and bound to the cell surface. Following PMA stimulation, GLS2 was removed from the membrane fractions and released to the extracellular culture medium [56].

The three cellular localizations identified so far, as well as several structural motifs that could enable interactions with other proteins, suggest that GLS2 is a multifaceted protein, which may be involved in other functions besides the generation of Glu [62]. Given the cell surface localization, it cannot be ruled out that this protein could interact with macromolecular components and/or regulate Gln levels in the extracellular space. Furthermore, GLS2 could be one enzyme that controls in situ Gln levels in the nucleoplasm and, hence, indirectly modulates the expression of Gln-regulated genes. This hypothesis is supported by the observation that nuclear GLS2 is catalytically active [59]. Additionally, it is also possible that GLS2 forms part of a transcriptional complex as a co-regulator. The few reports indicating the influence of ectopic *GLS2* expression on the transcription process will be discussed in detail in Section 4. Finally, as mentioned above, GLS2 has been shown to interact with two PDZ domain-containing proteins: α1-syntrophin (SNTA1) and a glutaminase-interacting protein (GIP) [61]. SNT1A belongs to a family of membrane-associated adaptor proteins and is involved in cytoskeletal organization and localization and/or regulation of many transduction proteins [65]. GIP, also known as Tax-interacting protein 1 (TIP-1), plays pivotal roles in many aspects of cellular signaling, protein scaffolding, and the modulation of tumor growth [66]. Although their significance remains unknown, interactions between GLS2 and GIP or SNTA1 strengthen the hypothesis that GLS2 is a moonlight protein, i.e., one that carries out multiple unrelated functions in a cell or organism [67].

Regardless of its potential regulatory functions, GLS2 is primarily an enzyme that plays a key role in the altered metabolism of cancer cells. Recently, the structure of the human glutaminase domain of the GLS2 protein, and the functional characterization of the residues critical for its activity, was described [68]. The glutaminase domain comprises residues Ile154 to Gly479, common to both the GAB and LGA isoforms. Accessibility to the active site is controlled by two loops: the “lid” and “activation” loop, the latter being a drug target [68]. It should be noted that GLS2 and GLS proteins are highly similar in their catalytic sites. Despite this similarity, several compounds have been identified that inhibit the activity of specific glutaminase isoforms. Some of them have exhibited promising antitumor properties in different types of cancer [69]. Clearly, inhibition of GLS2 activity may reduce the survival of those cells in which GLS protein acts as a tumor promoter. It should be emphasized that, in some cancer types, GLS2 seems to play a tumor suppressor role. These two faces of GLS2 isoforms, tumor promoter and tumor suppressor, in cancer pathogenesis, will be discussed below.

## 3. GLS2 as a Promoter of Tumorigenesis

The analysis of data from the Oncomine database has revealed the overexpression of *GLS2* compared to non-tumorigenic tissues in bladder, colon, rectum, head-and-neck, peritoneum, and lung cancers [70]. Among them, the latter is the one in which the potential role of GLS2 is, so far, best documented. An increased level of *GLS2* transcript and protein was detected in epidermal growth factor receptor (EGFR)-mutated non-small cell lung cancer (NSCLC) tissues compared to EGFR wild-type tumors [68]. It is worth mentioning that EGFR-tyrosine kinase inhibitors (EGFR-TKI) prolong progression-free survival in NSCLC patients, but many patients eventually develop disease progression due to TKI-acquired resistance [71]. A recent study has indicated that Gln is a critical nutrient in acquired-EGFR-TKI-resistant lung cancer cell lines, which show higher Gln dependency compared with their parental cells [72]. Interestingly, the silencing of *GLS2* diminished the viability of Gln-independent lung squamous-cell carcinoma QG56 cells and suppressed the activity of the mammalian target of rapamycin complex 1 (mTORC1) [73]. mTORC1 is a protein complex in which mTOR, a phosphoinositide 3-kinase-related protein kinase, plays a catalytic role. mTORC1 promotes translation, ribosome biogenesis, and autophagy and, thus, regulates cell growth. It acts as an effector of further oncogenic pathways, i.e., the Ras/Raf/Mek/Erk or PI3K/Akt pathways, which are often changed in the course of cancer which, in turn, results in the hyperactivation of mTORC1. In light of this, mTORC1 (and generally mTOR) inhibitors are promising cancer drugs [74]. In this context, GLS2 seems to exert a pro-oncogenic function through the regulation of mTORC1 signaling.

In the other model of NSCLC, A549 cells, *GLS2* silencing decreased the cell proliferation; a similar effect was observed when the cells were treated with an alkyl benzoquinone 1-(5-methoxy-3,6-dioxocyclohexa-1,4-dien-1-yl)pentadecan-2-yl acetate (AV-1) isolated from *Ardisia virens*, which preferentially inhibits GLS2 rather than GLS enzymes [75]. These authors have also found that, in human hepatoma HepG2 cells, AV-1 induces autophagy via the activation of AMP-activated protein kinase (AMPK) and inhibition of the phosphatidylinositol 3-kinase (PI3K)/AKT pathway which, in turn, suppresses mTORC1 activity. It should be noted that, in this study, *GLS2* silencing decreased the proliferation of HepG2 cells, which contrasts with several other papers documenting the tumor-suppressive role of GLS2 in hepatoma. All these reports will be presented in the following section of this review.

Xiao and colleagues reported a pro-oncogenic function of GLS2 in neuroblastoma [43]. In this study, an elevated expression of *GLS2*, and a reduced expression of *GLS*, was detected in the *MYCN*-amplified group when compared with non-amplified tumors. High *GLS2* expression was significantly correlated with poor survival, while *GLS* expression was negatively associated with the prognosis of neuroblastoma patients. The induction of *MYCN* expression in SHEP MYCN-ER neuroblastoma cells, which carry a single copy of the *MYCN* gene, led to a time-dependent activation of *GLS2* but not *GLS*. This finding indicates that N-Myc selectively induces *GLS2* expression in *MYCN*-amplified neuroblastoma cells [43]. It should be emphasized that *MYCN*, a member of the MYC proto-oncogene family, is overexpressed in many types of cancer, including neuroblastoma, and is responsible for promoting proliferation and the survival of tumor cells [76]. Thus, N-Myc-dependent *GLS2* activation may result in the reprogramming of cellular metabolism to maintain the viability and anaplerosis of the TCA cycle, ultimately leading to the Gln dependence of neuroblastoma cells. Indeed, further analyses have revealed that *GLS2* silencing substantially reduced proliferation and clonogenic potential in two *MYCN*-amplified neuroblastoma cell lines, Kelly and BE-2C, and attenuated their ability to form tumors in vivo. The silencing of *GLS2* induced apoptosis, which was suppressed when the cells were treated with dimethyl α-KG, a cell-permeable α-KG analog, indicating that these cells rely on GLS2-mediated glutaminolysis to refill TCA cycle intermediates necessary for proliferation. Furthermore, compared to the controls, *GLS2*-silenced cells presented diminished Gln consumption and Glu production, as well as decreased α-KG and adenosine triphosphate (ATP) generation. In addition, *GLS2* depletion decreased GSH content and increased the level of reactive oxygen species (ROS), but also inhibited aerobic glycolysis. Collectively, the results noted above indicate that GLS2 co-ordinates both Gln-dependent anaplerosis and aerobic glycolysis to sustain the proliferation and survival of *MYCN*-amplified neuroblastoma cells [43].

Cervical cancer is another malignancy in which the expression of *GLS2* is upregulated. Xiang and colleagues performed an immunohistochemical analysis of 58 cases of radioresistant tumor tissues and 86 cases of radiosensitive tumor tissues as well as 15 cases of adjacent normal cervical tissues [77]. A lack of GLS2 staining was found in the normal tissues, while cancer tissues contained considerable amounts of this protein. Notably, the GLS2 level in the tumor tissues of radioresistant patients was much higher than in those of radiosensitive patients. Similarly, GLS2 content was elevated in radioresistant cervical cancer cells HeLaR compared to the parental HeLa cells. *GLS2* silencing decreased the production of GSH and the other molecules involved in the cellular antioxidant defense, including nicotinamide adenine dinucleotide hydrogen (NADH) and nicotinamide adenine dinucleotide phosphate hydrogen (NADPH). It also increased the level of intracellular ROS, which translated into the enhanced radiosensitivity of HeLaR cells in vitro and in vivo [77]. These results corroborate the above-mentioned findings, indicating the role of GLS2 in the modulation of redox homeostasis in neuroblastoma [43].

The other neoplasm in which GLS2 plays a pro-oncogenic role is in luminal-subtype breast cancer. Analysis of data from the Cancer Genome Atlas (TCGA) has shown substantially higher levels of *GLS2* mRNA in luminal A (LumA) and luminal B (LumB) tumors than in basal and human epidermal growth factor receptor-2 (HER2^+^) tumors. Furthermore, receptor-positive breast tumors, the majority of which belonged to the luminal subtype, displayed significantly elevated level of GLS2 protein in comparison with normal tissue. In contrast, only a few receptor-negative breast cancer tissues, classified as basal subtype, contained large amount of this protein. A similar expression pattern was observed in breast cancer cell lines, with luminal-subtype cells showing high GLS2 content and basal-subtype cells showing low [47]. The level of GLS2 protein in breast cancer cell lines correlated with that of the GATA3 transcription factor; a positive correlation between the *GLS2* and *GATA3* transcript levels was found in human breast cancer tissues. Functional studies using MDA-MB-453 and T-47D luminal-subtype cells demonstrated that GATA3 binds to the promoter of the *GLS2* gene and drives its expression [47]. Given that GATA3 regulates luminal cell differentiation in the mammary gland [78], a high expression of *GLS2* may be inextricably linked to the luminal phenotype. Interestingly, in basal-subtype breast cancer cells, *GLS2* expression proved to be repressed by promoter methylation [47]. The same group documented a mitochondrial localization of GLS2 in MDA-MB-453 cells, wherein this enzyme was metabolically active and critical for glutamine-mediated anaplerosis. Abrogation of *GLS2* expression using shRNA significantly inhibited the proliferation of luminal-subtype cells in vitro and in vivo, but only slightly reduced this parameter in basal-subtype cells. On the contrary, neither *GLS* silencing, nor treatment with GLS-selective inhibitors, such as bis-2-(5-phenylacetamido-1,3,4-thiadiazol-2-yl)ethyl sulfide (BPTES) or CB-839 (Telaglenastat), affected the proliferation of luminal-subtype cells; however, these interventions decreased proliferation in basal-subtype cells. It should be noted that supplementation with dimethyl α-KG reversed the effect of *GLS2* or *GLS* knockdown, indicating that diminished proliferation resulted from disturbed TCA cycle anaplerosis. Notably, this study revealed that a small molecule, 968, a GLS inhibitor, targeted the activity of both GA isozymes with a moderate selectivity for GLS2. Treatment with this compound suppressed TCA cycle anaplerosis and proliferation in BPTES-resistant breast cancer cells; moreover, it reduced the tumorigenic potential of MDA-MB-453 cells, supporting a view of GLS2 as a druggable target in luminal-subtype breast cancers [47].

In a near-simultaneously published study, Dias and colleagues detected considerable amounts of GLS2 protein in 14 triple-negative (TN) breast cancer cell lines, which included basal, luminal, and mesenchymal subtypes, while a significantly lower content of GLS2 was found in four non-TN cell lines [79]. Interestingly, most cell lines also expressed GLS in addition to GLS2, but the lowest overall GA levels were detected in non-TN cell lines. TN cells lines presented higher GA activity and sensitivity to Gln withdrawal, which translated into increased sensitivity to CB-839 treatment compared to non-TN cells. *GLS2* knockdown diminished cell proliferation and increased oxidative stress. Similarly, either pharmacological or genetic GLS inhibition reduced proliferation, induced oxidative stress, and impaired TCA anaplerosis, while an ectopic expression of *GLS2* rescued this phenotype. Furthermore, in comparison with the control cells, the cells overexpressing *GLS2* presented an elevated invasion capacity as well as vimentin and actin stress fibers considered to be markers of the epithelial-to-mesenchymal transition (EMT), a process that plays a key role in cancer progression [80]. Finally, the authors examined the role of GLS2 in an in-vivo model in which MDA-MB-231 cells, transduced with either a *GLS2*-expressing or empty vector, were injected into mice. Astonishingly, the pro-tumorigenic effect, assessed as tumor volume and the number of lung metastases, was more pronounced when *GLS2* overexpression was accompanied by *GLS* knockdown. It is noteworthy that, in three cohorts of breast cancer patients, the expression of *GLS2* was significantly higher in tumors than in normal tissues; analysis of publicly available datasets (TCGA and the International Cancer Genome Consortium) revealed a correlation between high *GLS2* expression and decreased overall, disease-free, and distant metastasis-free survival [79]. By contrast, when the TCGA pan-cancer patients with five years of follow-up were stratified into those who express and those who do not express *GLS2*, the former group had a higher survival rate than the latter [81]. This discrepancy, probably resulting from differences in the criteria used, hints at the complexity of the role of GLS2 in breast cancer, which is largely due to the heterogeneity of these tumors. In fact, while the paper by Dias and colleagues clearly indicates that *GLS2* overexpression elevates mesenchymal markers in several breast cancer cell lines [79], a study by Ramirez-Peña and colleagues demonstrated that *GLS2* expression is inversely correlated with EMT in breast cancer [81]. Firstly, analysis of the breast cancer patient samples from TCGA revealed an inverse correlation between *GLS2* and EMT-signature gene expression. While the highly metastatic basal subtype was enriched with *GLS2* deletions, the less aggressive subtypes were enriched in amplifications of these genes. Furthermore, an inverse correlation between *GLS2* and EMT has also been observed in experimental models. The level of the *GLS2* transcript was lower in mesenchymal breast cancer cell lines than in epithelial cells. Immortalized human mammary epithelial HMLE cells, genetically modified to express EMT-inducing transcription factors, which results in differentiation into the mesenchymal state, presented a diminished expression of the *GLS2* gene compared to the control epithelial cells. This suppression of *GLS2* correlated with decreased mitochondrial activity and Gln dependence. The inhibition of EMT restored *GLS2* expression and Gln utilization. Additionally, an ectopic expression of *GLS2* in SUM159 mesenchymal breast cancer cells enhanced mitochondrial activity and Gln consumption; it also inhibited mammosphere formation, which is a component of cancer-stem-cell potential, although it did not affect the proliferation of these cells. The findings of this study indicate that epithelial breast cancer cells rely on Gln and that cells that undergo EMT become Gln independent. Furthermore, EMT inhibition results in an elevated *GLS2* expression and a subsequent metabolic shift in mesenchymal cells, which may sensitize them to chemotherapy [81]. The results of experimental studies documenting the tumor-promoting properties of GLS2 are summarized in Table 1.

The pro-oncogenic functions of GLS2 discussed above indicate that this protein may serve as a valuable therapeutic target in some cancers; however, this idea has not received much attention until recently, and the overwhelming majority of research has focused on targeting GLS isoforms. In this context, both Gln analogues and small molecule allosteric inhibitors have been tested. Gln analogs, such as 6-diazo-5-oxo-L-nor-leucine (DON), acivicin, or azaserine, have demonstrated anticancer activity in vitro and in vivo, but failed in the early phases of clinical trials due to side effects resulting from a lack of selectivity. It is worth mentioning that Gln analogs inhibit other enzymes that hydrolyze this amino acid, which results in off-target effects. The possibility of specific GA inhibition has advanced significantly with the development of allosteric inhibitors which, as already mentioned, are now mainly directed against GLS isoforms; the most promising is CB-839, which is currently being tested in several clinical trials. The current state of knowledge about the inhibitors of Gln metabolism have been discussed comprehensively in two very recent reviews [69,82]. The following section is limited to the description of the allosteric inhibitors that exhibit at least partial specificity for GLS2 (Table 2).

To date, the only known molecular scaffolds that potently and selectively target GLS2 are alkyl benzoquinones, isolated from *A. virens*. Among them, the above-mentioned AV, also known as ardisianone, shows high selectivity against GLS2 over GLS, with a half-maximal inhibitory concentration (IC50) of 0.28 μM for GLS2 and 2.1 μM for GLS [75]. The use of homologous modeling, docking, and mutagenesis studies have identified a potential binding site for AV-1 in GAB as well as residues crucial for the selectivity of this compound towards GAB. It is worth noting that two other natural alkyl benzoquinones, referred to as AV-2 and AV-8, exhibited only slightly higher IC50 values for GLS2 and GLS than AV-1. The keto/hydroxyl substituents at positions 1 and 4 in the benzoquinone core, as well as the acetate group at position 2, proved to be essential for potency towards each of the enzymes [75]. AV-1 exhibited anticancer properties against human hormone-refractory prostate cancer cells PC-3 and DU-145 [83] and acute myeloid leukemia cells HL-60 [84]. None of the studies noted above, however, have analyzed AV-1’s potential to inhibit the enzymatic activity of GA isoforms.

Another compound showing an ability to inhibit the catalytic activity of GLS2 is 986, a member of the benzophenanthridinone family, which was originally identified as a GAC inhibitor with anticancer properties in human breast cancer cells MDA-MB231 and SKBR3, as well as in a B-cell lymphoma model in vitro and in vivo [27]. In a more recent study, 968 was found to be a pan-GA inhibitor, with threefold selectivity for GLS2 over GLS [47]. This compound showed anti-proliferative activity in several breast cancer cell lines, including DU4475 cells that display intrinsic resistance to BPTES, a selective GLS inhibitor [85]. Consistent with the partial selectivity of 968 for GLS2 over GLS, the inhibition of GLS2-mediated TCA cycle anaplerosis was more pronounced in MDA-MB-453 cells containing a significant amount of GLS2 than in MDA-MB-231 cells, in which only traces of this protein were found. Furthermore, contrary to BPTES, 968 inhibited the growth of tumors formed by MDA-MB-453 cells in mice [47]. Aside from the above-mentioned cancers, 968 displayed anticancer activity in NSCLC cells A549, H23, H1299, and Spc-A1 [86], ovarian cell lines HEY, SKOV3, IGROV-1 [87], endometrial cancer cells Ishikawa and HEC-1B [88], as well as HCC cells LM3 and, to a lesser extent, 7402 and HepG2 [89]. In all these studies, 968 was considered to perform as a GLS inhibitor, while its potential effect on GLS2 has not been investigated; however, considering the pro-oncogenic role of GLS2 in some types of cancer, e.g., lung cancer [73,75], it cannot be ruled out that the observed reduction in survival is, at least partially, also due to GLS2 inhibition.

Finally, a group of thiazolidine-2,4-dione derivatives exhibited inhibitory activity against both GLS and GLS2. They diminished the clonogenic potential of human pancreas adenocarcinoma AsPC-1 cells and reduced the tumor size formed by AsPC-1 in mice; however, even though some of them inhibited GLS2 (e.g., compound 6 had an IC50 value of 102 nM), they were more selective against GLS (IC50 value of 50 nM) [90], clearly indicating that further studies are required to develop GLS2-specific inhibitors.

## 4. GLS2 as a Suppressor of Tumorigenesis

Twenty years ago, Turner and McGivan documented, for the first time, co-expression of the *GLS* and *GLS2* genes in a single cancer cell type. The results of the northern blot analysis allowed the authors to speculate that the level of *GLS2* mRNA was consistently higher in slow-growing colorectal adenoma AA/C1 cells than in the rapidly proliferating colorectal carcinoma HT29 cells [55]. Subsequently, Perez-Gomez and colleagues have confirmed the co-expression of both genes in tumor cells of different origin. According to their study, HepG2 cells contain much more *GLS2* transcript than *GLS* although, at the protein level, this difference is not apparent [17]. The results of the measurement of the enzymatic activity indicates that GLS protein, not GLS2, is mainly responsible for GA activity in HepG2 cells, which is consistent with a previous report [91]. Taking into account that normal, non-proliferating hepatocytes express *GLS2* [35], it is possible that *GLS* isoforms become upregulated during malignant transformation. The same group detected both *GLS* and *GLS2* transcripts in human leukemia cells, but only the latter transcript in mature, non-proliferating lymphocytes from the medullar blood of a patient suffering aplasia. Based on these results, the authors hypothesized that *GLS2* expression is a characteristic feature of resting or quiescent cells, while the overexpression of *GLS* is associated with elevated proliferation rates [17].

As mentioned in Section 3, Lee and colleagues documented the inhibitory effect of *GLS2* silencing on the proliferation of HepG2 cells derived from hepatocellular carcinoma (HCC) [75]; nevertheless, this is one of the few reports, if not the only one, suggesting a pro-oncogenic role for GLS2 in this tumor type. Indeed, the immunohistochemical analysis of the GLS2 level in 112 HCC and 111 adjacent non-tumor tissues (NT) revealed significantly lower GLS2 staining in tumors than in NT tissues; a lack of GLS2 staining correlated with a short survival time [23]. Interestingly, an inverse pattern in GLS staining was also observed, with an elevated level of this protein in HCC, which indicates that GLS and GLS2 play opposing roles in HCC. Furthermore, the authors examined the expression of both GA isoforms in a set of liver tissues mimicking HCC transformation. The intensity of GLS2 staining was high in NT and low-grade fibrotic liver tissues, and low in HCC. On the contrary, GLS staining was weak in NT tissues but gradually increased in parallel with disease progression, at its most intense in HCC [23]. These findings allow for speculation that, during HCC transformation, Gln metabolism switches from GLS2- to GLS-dependent; they are consistent with an earlier study on MYC-induced mouse liver tumors [92].

Several other reports have confirmed diminished *GLS2* expression in HCC compared to healthy tissues [39,44,45,93] and pointed to promoter methylation as the mechanism responsible for this phenomenon [44,45]. Furthermore, numerous studies have documented the onco-suppressive function of GLS2 in multiple HCC models in vitro and in vivo. For instance, an ectopic expression of *GLS2* decreased the ability of HepG2 cells to form colonies. On the other hand, ablation of *GLS2* increased the production of reactive oxygen species (ROS) and sensitized HepG2 cells to H_2_O_2_-induced apoptosis; it also diminished the α-ketoglutarate and ATP levels and oxygen consumption. Notably, the *GLS2* transcription in HepG2 cells proved to be induced by p53 [39], a tumor suppressor whose role in the prevention of HCC has been widely documented [94]. It is therefore tempting to speculate that, as a p53 target, GLS2 might contribute to the cancer-suppressive properties of p53 in HCC. In another study, the overexpression of *GLS2* markedly inhibited the growth of xenograft tumors formed by two HCC cell lines, Huh1 and Huh7, compared with tumors formed by the control cells transduced with an empty vector. Conversely, the knockdown of *GLS2* in PLC/PRF/5 HCC cells containing traces of the *GLS2* transcript significantly enhanced anchorage-independent cell growth and the formation of tumors in vivo. A subsequent functional analysis revealed that GLS2 negatively regulates the PI3K/AKT signaling pathway, which contributes to the tumor-suppressive properties exerted by GLS2 in HCC [45]. In another study, GLS2 proved to be critical for the inhibition of HCC cell migration and invasion [93]. Firstly, a negative correlation between the amount of GLS2 protein and the ability of different HCC cell lines to migrate and invade was observed. Secondly, an ectopic expression of *GLS2* in Mahlavu and Huh7 cells, which contain barely detectable endogenous GLS2 protein, diminished migration and invasion; the opposite effect was obtained when *GLS2* was silenced in HCC36 and HepG2 cells containing a considerable amount of GLS2. Further analysis provided compelling evidence that GLS2 represses EMT [93]. Compared to the controls, Mahlavu cells in which *GLS2* was overexpressed presented more epithelial morphology, an elevated level of epithelial marker E-cadherin, and lowered levels of mesenchymal markers, vimentin, and fibronectin; the opposite effect was obtained by *GLS2* silencing in HepG2 cells. The aforementioned modification diminished the level of Snail, a transcription factor which plays a crucial role in tumor metastasis [95]. It is particularly worth noting that overexpression of SNAI1, the gene encoding Snail, reversed the GLS2-mediated phenotype in vitro, and restored both intrahepatic and lung metastasis inhibited by GLS2 in the orthotopic model. In addition, further mechanistic studies have shown that GLS2 functionally binds to DICER, a protein involved in the production of miRNAs [96], in a non-enzymatic activity manner. This interaction stabilizes DICER and promotes the maturation of miR-34a which, in turn, inhibits SNAI1 expression [93]. Notably, DICER is a central player in the miRNA and RNAi pathways, and alterations in its level and activity can lead to several diseases, including cancer, while the promotion of its function may be beneficial [97]. Clearly, further investigations are needed to elucidate whether DICER stabilization by GLS2 can also modulate other miRNAs/RNAi, as well as how it influences the cancer cell phenotype.

Yet another protein that interacts with GLS2, at least in HCC cells, is Ras-related C3 botulinum toxin substrate 1 (Rac1), a member of the Rho GTPase family, involved in the regulation of tumor angiogenesis, invasion, and metastasis; these processes underlie malignant transformation. Rac1 hyperactivation and overexpression correlates with a malignant phenotype in several tumors of different origin [97]. The interaction between GLS2 and Rac1 has been observed not only in Huh-1 cells transduced with vectors expressing *GLS2* and *RAC1*, but also in wild-type Huh-1 and HepG2 cells [98]. Interestingly, the C-terminus of GLS2, which does not contain the GA catalytic domain, proved to be necessary and sufficient for the interaction between this protein and Rac1. GLS2 binding inhibits the interaction of Rac1 with its activators, Tiam1 and VAV1. This negative regulation of Rac1 contributed to the inhibitory effect of GLS2 on the migration and invasion of several HCC cells in vitro as well as on their metastatic potential in vivo. Last but not least, the finding of this study is that GLS2 mediates the role of p53 in inhibiting metastasis. The silencing of *GLS2* or *TP53* markedly elevated the migration and invasion of Huh-1 and HepG2 cells in vitro and their metastasis in mice, whereas the simultaneous knockdown of both genes did not present an additive effect on either the migration/invasion of these cells or their ability to metastasize. Furthermore, a single knockdown of either *GLS2* or *TP53* significantly activated Rac1, while their simultaneous silencing did not present an additive effect on the activity of Rac1. The overexpression of *GLS2* abolished the promoting influence of TP53 knockdown on Rac1 activity, suggesting that GLS2 mediates p53 function in inhibiting Rac1 activity [98]. It should be emphasized that Rac1 is a pleiotropic regulator of many cellular processes (i.e., proliferation, ROS-mediated cell death, and cell cycle progression), and has multiple effectors [97]. It is therefore tempting to speculate that the interaction between GLS2 and Rac1, which inhibits Rac1 activity, modulates the action of many molecules (not yet identified), which translates into the suppression of the malignant phenotype.

In a very recent study, Suzuki and colleagues not only confirmed the tumor-suppressive activity of GLS2 in HCC, but also shed new light on the mechanism underlying this phenomenon [99]. By 120 weeks of age, all *Gls2* knockout mice included in the experiment developed either B-cell lymphomas, HCC, or both tumor types. Conversely, none of the wild-type mice exhibited any tumor. In addition, the animals were subjected to the hepatocarcinogenic Stelic Animal Model (STAM) protocol, which reflects non-alcoholic steatohepatitis closely associated with HCC [100]. A lack of *Gls2* resulted in the earlier development of HCC tumors that were larger in size than those produced in wild-type mice. Compared to WT-STAM animals, KO-STAM mice displayed a lowered level of ferroptosis markers in both normal liver and HCC. Furthermore, the level of malondialdehyde, an indicator of ferroptosis, was reduced in the livers of knockout mice compared with wild-type mice; hepatocytes from knockout animals proved to be less sensitive to inducers of ferroptosis. This resistance to ferroptosis was attenuated by an ectopic expression of *Gls2*, indicating that GLS2 is necessary for the induction of ferroptosis in hepatocytes. These findings extend to human HCC cells, in which modifications of GLS2 levels resulted in a correspondingly altered extent of ferroptosis. GLS2 enhanced the production of lipid ROS through the conversion of Glu to α-KG which, in turn, promoted ferroptosis. Likewise, compared to the controls, *GLS2*-overexpressing cells formed significantly smaller tumors after injection into the severe combined immunodeficient (SCID) mice, which was accompanied by an increase in the expression of ferroptosis markers. Notably, in this study, the GA core domain, located between positions 177 and 463, was necessary for the tumor-suppressive functions of GLS2 and its ability to promote ferroptosis [99]. Interestingly, the enzymatic activity of GLS2 may be regulated by the general control of amino acid synthesis 5-like 1 (GCN5L1) protein, as demonstrated in mice hepatocytes. Mitochondrial-enriched GCN5L1 suppresses, while the absence of GCN5L1 increases GLS2 activity; this modulation is mediated in part by the change in acetylation of lysine 279, located in the putative catalytic domain of GLS2. The activation of GLS2 promotes hepatic glutaminolysis, which further leads to the activation of mTORC1 signaling [101]. The same mode of regulation of the enzymatic activity of GLS2 was observed in HCC. The deletion of GCN5L1 enhances Gln metabolism and promotes HCC cell proliferation and mTORC1 activity. Mechanistically, GCN5L1 promotes the acetylation and inactivation of both GLS2 and GLS, and increases enzyme oligomerisation. It is worth noting that an inverse correlation has been observed between GCN5L1 and mTORC1 levels and GA activity in HCC patients [102].

Collectively, the overwhelming majority of data collected to date clearly indicate that, in HCC, GLS2 is a bona fide tumor suppressor. This conclusion comes from observations made on several cohorts of patients as well as from numerous in vitro and in vivo studies, which have shed new light on the mechanism of GLS2 action. It is worth noting that GLS2 seems to inhibit tumorigenesis in other types of gastrointestinal cancer. Thus, *GLS2* expression proved to be diminished in colon tumor tissues compared to adjacent non-tumor tissues and, as in the case of HCC, promoter methylation was found to be a key mechanism underlying *GLS2* silencing in colon cancer. Even though the analysis was performed on a small number (n = 5) of tissues, and the level of GLS2 protein was not examined, the results of studies on human colon cancer cells (HCT116) revealed that an ectopic expression of *GLS2* markedly reduced the viability and number of cell colonies. Additionally, an ectopic expression of GLS2 led to a significant increase in the number of cells in the G2/M phase and a reduction of the levels of p21 and cyclin D1 [44]. More recently, the downregulation of *GLS2* expression was noted in a cohort of 36 gastric cancer tissues compared to the adjacent non-cancer tissues. *GLS2* overexpression suppressed the viability of gastric cancer MGC-803 cells and their ability to migrate; it also induced apoptosis [103]. Both of the studies noted above were performed on only one cell line and do not include functional studies; therefore, further in-depth analysis is necessary to elucidate the significance of reduced *GLS2* expression in colon and gastric cancer.

Another type of neoplasm in which GLS2 appears to exert suppressive properties is glioblastoma, an astrocytic tumor with the highest malignancy grade according to WHO classification (WHO IV) [104]. *GLS2* expression in these tumors is abolished or significantly lower than in non-tumorigenic brain tissues, which was first demonstrated by examining groups of several patients [16,46] or by analyzing data from TCGA [70]. Nevertheless, a similar observation was also made in a recent study conducted on a relatively large cohort of 153 patients with astrocytomas of different grades of malignancy. Compared to non-tumorigenic brain tissues, pilocytic astrocytomas (WHO grade I) display a significantly lower expression of *GLS2*, which is further reduced with an increasing grade of malignancy [105]. Notably, promoter methylation has proved to be a crucial mechanism responsible for the downregulation of *GLS2* in glioblastoma [46]. Interestingly, the expression of *GLS* is upregulated in glioblastoma compared to non-tumorigenic brain tissues [16], reinforcing the idea that GLS and GLS2 play opposing roles in certain cancer types. An ectopic expression of *GLS2* decreased the viability and proliferation of glioblastoma T98G, U87MG and LN229 cells, as well as their ability to form colonies and migrate [106,107]. Microarray analysis revealed alterations in the expression of several genes, some of which are relevant to malignancy. One of the downregulated genes, *MGMT*, codes for the repair protein O^6^-methylguanine-DNA methyltransferase (MGMT), which reverses the alkylation process, thereby contributing to resistance to the alkylating agent temozolomide (TMZ), used in chemotherapy. Since DNA methylation contributes to *MGMT* silencing, the methylation status of the *MGMT* promoter is considered to be a biomarker of a patient’s response to TMZ treatment [108]. Diminished *MGMT* expression in T98G cells transfected with *GLS2* translated into a decreased level of MGMT protein and activity which, in turn, resulted in increased sensitivity to TMZ. The overexpression of *GLS2* did not change the methylation status of MGMT; therefore, the mechanism by which GLS2 diminishes *MGMT* transcription remains unknown [109]. Similar to T98G cells, two other glioblastoma cell lines, U87MG and LN229, also became more sensitive upon transfection with the *GLS2* sequence. Given that U87MG cells do not express *MGMT* [110], a mechanism unrelated to the modulation of the MGMT level must have been responsible for the increase in sensitivity to TMZ. It could, for example, be related to oxidative stress induced by TMZ [111]. Indeed, an ectopic *GLS2* expression sensitized all three cell lines to treatment with oxidate agents hydrogen peroxide (H_2_O_2_) and arsenic trioxide (ATO) [29,107]. Upon treatment with H_2_O_2_, *GLS2*-transfected cells presented a reduction in the phosphorylation of PDK1, PI3K, and AKT. Pre-treatment with PDGF-BB, an activator of AKT phosphorylation, prevented H_2_O_2_-evoked cell death in *GLS2*-transfectants, indicating that the downregulation of the PI3K/AKT pathway contributes to increased sensitivity to H_2_O_2_ [107]. Notably, both T98G and LN229 cells transfected with *GLS2* displayed a reduced activity of catalase (CAT). Additionally, a diminished activity of superoxide dismutase (SOD) was found in LN229 transfectants, while it was elevated in *GLS2*-transfected T98G cells. Furthermore, total antioxidant capacity increased and lipid peroxidation decreased in T98G and LN229 cells upon transfection with *GLS2*, supporting the antioxidant effect of GLS2 [112]. It is noteworthy that this study identified a series of miRNAs whose expression was changed by an ectopic expression of GLS2. Thus, miRNA-140-3p, miRNA-1246, miRNA-1260a, miRNA-21-5p, and miRNA-146a-5p were overexpressed in both T98G and LN229 after an overexpression of *GLS2*. One more miRNA, miRNA-92a-3p, was additionally upregulated in LN229 cells transfected with *GLS2*. Although further functional analysis is necessary to clarify the significance of the changes described above in the miRNome of *GLS2*-transfected cells, it is worth noting that the included molecules have been linked to carcinogenesis in different tumor models [112].

Interestingly, relative to the controls, T98G cells transfected with the *GLS2* sequence presented diminished proliferation when cultured in a medium containing adult bovine serum [58]. Such a procedure makes it possible to avoid non-physiological levels of cysteine in the culture medium, which induces increased reliance on Gln anaplerosis [113]. Compared to WT T98G cells, *GLS2*-transfected T98G cells showed an increased level of p53 in nuclei and decreased level of c-Myc in cytoplasm. Likewise, an elevated level of GLS2 observed in the nuclei of PMA-treated neuroblastoma SH-SY5Y cells was accompanied by significantly increased amounts of p53, p21, and cell cycle arrest at the G2/M stage. Since the treatment of SH-SY5Y cells with PMA reduces their proliferation, as does the transfection of T98G cells with the *GLS2* sequence, it is tempting to speculate that the nuclear localization of GLS2 plays an important role in the inhibition of proliferation and is mediated, at least partially, by p53-dependent mechanisms [58].

As mentioned above, an ectopic *GLS2* expression sensitized glioblastoma cells to treatment with TMZ [107]. In a very recent study, Luo and colleagues showed that TMZ-resistant glioblastoma cells (U251TR) contain a lesser amount of GLS2 than U251 cells [53]. An ectopic *GLS2* expression in U251TR cells diminished proliferation and migration, and both parameters were further decreased upon TMZ treatment. This modification increased the lipid ROS level, which was reversed by Ferrostatin-1, an inhibitor of ferroptosis; it also increased the level of E-cadherin and decreased the level of vimentin. These results suggest that GLS2 can drive ferroptosis and repress EMT, which is consistent with the previous observations made in HCC cells [93,99]. Similar results were obtained in the intracranial tumor model, indicating, for the first time, that GLS2 exerts anti-glioma effects in in vivo settings [53].

In this regard, it is worth noting the report by Kim and colleagues documenting the correlation between the level of GLS2 and the regional heterogeneity of 5-aminolevulinic acid (5-ALA) fluorescence in glioblastoma [114]. Fluorescence-guided surgery using 5-ALA has been introduced in the treatment of high-grade gliomas, as it enables the intraoperative visualization of malignant tissue and differentiates it from normal brain [115]. Inter- and intra-tumoral molecular, cellular, and metabolic heterogeneity significantly contributes to therapy failure in glioblastoma [116]. Interestingly, the 5-ALA fluorescence intensity in glioblastoma shows regional heterogeneity, which may result from differences in cell density and/or intra-tumoral metabolic heterogeneity [117]. Kim and colleagues identified *GLS2* as a key gene in the regulation of 5-ALA metabolism in glioblastoma. Each of the three glioblastoma cell lines used in this study, namely T98G, U87MG, and LN18, transduced with a *GLS2* sequence, displayed significantly attenuated fluorescence intensity after 5-ALA treatment compared to their WT counterparts. The ratios of NADPH/NADP and GSH/GSSG were significantly reduced after 5-ALA treatment, and *GLS2* expression elevated the levels of NADPH/NADP, which was most likely linked with an increased capacity for 5-ALA metabolism. Furthermore, 5-ALA fluorescence was inversely correlated with *GLS2* expression and the NADPH level in glioblastoma tissues. Collectively, these results allow for a conclusion that regional metabolic heterogeneity in glioblastoma leads to low *GLS2* expression and low levels of NADPH and GSH levels in the malignant area, while high *GLS2* expression, together with high levels of NADPH and GSH, is found in the lower grade area [114]. The results of the above experiments, which suggest that GLS2 behaves as a tumor suppressor, are summarized in Table 3.

## 5. Conclusions and Future Directions

Impaired metabolism is generally considered to be a hallmark of cancer. Increased Gln uptake and enhanced glutaminolysis are key metabolic features of many cancers. Consequently, neoplasms of different origin display an elevated expression of GA, which correlates with the growth rate and malignancy of tumors. Targeting Gln, for instance through the inhibition of GA, appears to be a promising approach in treating Gln-addicted tumors.

In some cancers, *GLS2* expression is regulated by oncoproteins and clearly contributes to tumorigenesis by supporting cancer cell proliferation and tumor growth. Nevertheless, current knowledge revises the traditional view of the function of GLS2 as an enzyme that drives carcinogenesis; rather, it hints that this protein acts as a molecule-suppressing neoplastic phenotype. This appears to be particularly true for HCC. Recent studies have conclusively proven that GLS2 is a bona fide tumor suppressor at an organismal level, but mounting evidence indicates that a similar situation also occurs in glioblastoma. So far, single reports suggest that GLS2 protein may also inhibit the development of other cancers.

The precise mechanism underlying the tumor-suppressing function of GLS2 is yet to be fully elucidated, but it is believed to involve the regulation of cellular metabolism and redox balance. It should be emphasized that, in several research models, the suppressor role of GLS2 proved to be independent of the enzymatic activity of this protein. Although progress has been made in identifying the pathways triggered by GLS2, as well as its functional protein partners, there are still many unanswered questions. Future research should focus on determining whether the mechanism described in HCC is universal and also applies to other types of cancer; moreover, further work is needed to investigate the effects of simultaneous *GLS* silencing and *GLS2* overexpression. Extensive research in this area may pave the way for effective cancer treatment strategies.

## Figures and Tables

**Table 1:** Experimental studies indicating that *GLS2* acts as a tumor promoter.

Cancer	Model	Outcome	Ref.
**Effects of *GLS2* Inhibition**			
Non-small cell lung cancer (NSCLC)	A549 cells with *GLS2* knockdown	Decreased cell growth	[75]
Hepatocellular carcinoma (HCC)	HepG2 cells with *GLS2* knockdown or AV-1 treatment	Decreased cell growth, inhibited anchorage-independent colony formation, autophagy induction, P13K/Akt inhibition, suppressed mTORC1 activity	
Lung squamous-cell carcinoma (SCC)	QG56 cells with *GLS2* knockdown	Diminished viability, decreased mTORC1 activity	[73]
Neuroblastoma	*MYCN*-amplified Kelly and BE-2C cells with *GLS2* knockdown	Reduced proliferation and clonogenic potential, attenuated tumors forming ability, decreased GSH, α-KG and ATP level, increased ROS level, inhibited Gln-dependent anaplerosis of TCA cycle and aerobic glycolysis	[43]
Cervical cancer	HeLa and HeLaR cells with *GLS2* knockdown	Increased intracellular ROS levels, decreased production of NADH, NADPH, GSH, enhanced radiosensitivity	[77]
	mouse model injected with HeLa, HeLaR and HeLaR cells with *GLS2* knockdown	Enhanced radiosensitivity	
Luminal-subtype breast cancer	MDA-MB-453 cells with *GLS2* knockdown	Reduced proliferation, suppressed glutamine-mediated TCA cycle anaplerosis	[47]
	mouse model injected with MDA-MB-453 cells	Reduced tumorigenesis	
Triple-negative (TN) breast cancer	TN and non-TN cells with *GLS2* knockdown	Diminished cell proliferation, increased oxidative stress	[79]
	HMLE cells with *GLS2* knockdown	Gln independence, reduced mitochondrial activity	[81]
**Effects of *GLS2* Overexpression**			
Triple-negative (TN) breast cancer	TN and non-TN breast cancer cells overexpressing *GLS2*	Elevated invasion capacity	[79]
	mouse model injected with MDA-MB-231 cells	Pro-tumorigenic effect, decreased overall, disease-free and distant metastasis-free survival	
	SUM159 cells overexpressing *GLS2*	Enhanced mitochondrial activity, glutamine independence, inhibited mammosphere formation, higher intracellular GSH and ATP level, higher basal respiration, reduced cells viability	[81]

**Table 2:** Small molecule allosteric inhibitors showing at least partial specificity for GLS2.

Compound	Class	Selectivity	Anticancer Activity
AV-1 (ardisianone)	alkyl benzoquinone	approximately tenfold selectivity against GLS2 over GLS [75]	human hormone-refractory prostate cancer cells PC-3 and DU-145145 [83], acute myeloid leukemia cells HL-60 [84]
986	benzophenanthridinone	threefold selectivity against GLS2 over GLS [47]	breast cancer cell lines DU4475 [85], MDA-MB-453, MDA-MB-453 xenografts [47], NSCLC cell lines A549, H23, H1299, and Spc-A1 [86], ovarian cell lines HEY, SKOV3, IGROV-1 [87], endometrial cancer cell lines Ishikawa and HEC-1B [88], HCC cell lines LM3, 7402 and HepG2 [89]
derivative 6	thiazoli-dine- 2,4-dione	twofold selectivity against GLS over GLS2 [90]	human pancreas adenocarcinoma AsPC-1 cells AsPC-1 mice xenografts [90]

**Table 3:** Experimental studies indicating that *GLS2* acts as a tumor suppressor.

Cancer	Model	Outcome	Ref.
**Effects of *GLS2* Inhibition**			
Hepatocellular carcinoma (HCC)	HepG2 cell line	Increased ROS production, sensitization to H_2_O_2_-induced apoptosis, decreased oxygen consumption, α-ketoglutarate and ATP levels	[39]
	PLC/PRF/5 cell line with *GLS2* knockdown	Enhanced anchorage-independent cell growth and formation of tumors	[45]
	HepG2, HCC36 and Mahlavu cell lines with *GLS2* knockdown	Enhanced cell migration and invasion, diminished level of Snail, reduction of miR-34a expression	[93]
	Mice with *GLS2* knockout	Earlier development of larger tumors, resistance to ferroptosis, increased level of MDA and oxidative stress	[103]
**Effects of *GLS2* Overexpression**			
Hepatocellular carcinoma (HCC)	HepG2 cells overexpressing *GLS2*	Reduced cell colony formation	[39]
	Huh1 and Huh7 cells overexpressing *GLS2*	Reduced xenograft tumor growth, downregulation of PI3K/AKT signaling pathway	[45]
	PLC/PRF/5 cells with *GLS2* knockdown	Downregulation of PI3K/AKT signaling pathway	
	Mahlavu and Huh7 cells overexpressing *GLS2*	Decreased cell migration and invasion, stabilization of Dicer protein, induction of Dicer-dependent miR-34a maturation, repressed EMT, motility and metastasis	[93]
	Huh-1 cells co-transduced with *GLS2* and *RAC1*	Inhibited cell migration, invasion, metastasis and Rac1 activity	[98]
	HepG2, HepG3, SKHep1 cells overexpressing *GLS2*	Enhanced ferroptosis through αKG-dependent increase of lipid ROS	[99]
	Mice model injected with SKHep1 cells overexpressing *GLS2*	Reduced tumor formation, increased expression of ferroptosis markers	
Colon cancer	HCT116 cells overexpressing *GLS2*	Reduced viability and number of cell colonies, increased number of cells in G2/M phase, reduced levels of p21 and cyclin D1	[44]
Gastric cancer	MGC-803 cells overexpressing *GLS2*	Suppressed cell viability and ability to migration, induced apoptosis	[103]
Glioblastoma	T98G, U87MG and LN229 cells overexpressing *GLS2*	Decreased cells viability, proliferation, ability to form colonies and migration, increased TMZ and H_2_O_2_-mediated oxidative stress sensitivity, downregulation of the PI3K/AKT pathway	[106,107]
	T98G, SFxL and LN229 cells overexpressing *GLS2*	Inhibition of cell migration, decreased apoptosis, antioxidant status and cellular motility, H_2_O_2_ and ATO sensitivity	[29]
	T98G and LN229 cells overexpressing *GLS2*	Reduced CAT activity, increased total antioxidant capacity, decreased lipid peroxidation, miRNA-140-3p, miRNA-1246, miRNA-1260a, miRNA-21-5p and miRNA-146a-5p overexpression	[112]
	LN229 cells overexpressing *GLS2*	Diminished SOD activity, miRNA-92a-3p upregulation	
	U251 and U251TR cells overexpressing *GLS2;* intracranial U251TR model	Diminished cell proliferation and migration, increased lipid ROS and E-cadherin level, decreased vimentin level, repressed EMT	[53]
	T98G cells overexpressing *GLS2*	Decreased cell proliferation, increased level of p53 in nuclei, decreased level of c-Myc in cytoplasm	[58]
Neuroblastoma	SH-SY5Y cells treated with PMA	Inhibited proliferation, increased p53 and p21 level, cell cycle arrested at G2/M stage	
